# Association between urinary sodium excretion and uric acid, and its interaction on the risk of prehypertension among Chinese young adults

**DOI:** 10.1038/s41598-018-26148-3

**Published:** 2018-05-17

**Authors:** Yang Wang, Jia-Wen Hu, Peng-Fei Qu, Ke-Ke Wang, Yu Yan, Chao Chu, Wen-Ling Zheng, Xian-Jing Xu, Yong-Bo Lv, Qiong Ma, Ke Gao, Yue Yuan, Hao Li, Zu-Yi Yuan, Jian-Jun Mu

**Affiliations:** 1 Department of Cardiovascular Medicine, First Affiliated Hospital of Xi’an Jiaotong University, Xi’an, China; 2 Key Laboratory of Molecular Cardiology of Shaanxi Province, Xi’an, China; 3 Assisted Reproduction Center, Northwest Women and Children’s Hospital, Xi’an, China; 4 General Ward, Henan Provincial People’s Hospital, Zhengzhou, Henan China; 5 Department of Critical Care Medicine, First Affiliated Hospital of Medical School, Xi’an Jiaotong University, Xi’an, China

## Abstract

High uric acid (UA) level and high salt intake are reportedly associated with cardiovascular disease. This study investigated the association between UA and urinary sodium excretion, as well as its interaction on the risk of prehypertension. A total of 1869 participants without hypertension were recruited from a previously established cohort in Shaanxi Province, China. The participants were classified as normotensive or prehypertensive on the basis of their blood pressure. Increasing quartiles of sodium excretion were associated with high urinary UA/creatinine levels in prehypertensive participants. Estimated sodium excretion positively correlated with urinary UA/creatinine excretions in the prehypertensive group. In addition, the multivariate-adjusted odds ratios for prehypertension compared with normotension were 1.68 (1.27–2.22) for sodium excretion and 1.71 (1.21–2.42) for serum UA. Increasing sodium excretion and serum UA were associated with higher risk of prehypertension. Compared with the lowest quartiles, the highest sodium excretion and serum UA quartiles entailed 3.48 times greater risk of prehypertension. Sodium excretion is associated with urinary UA excretion in prehypertensive participants. The present study shows that high levels of salt intake and serum UA simultaneously are associated with a higher risk of prehypertension.

## Introduction

Hypertension contributes to the burden of heart disease, stroke, and kidney failure, and it is one of the leading causes of morbidity and mortality. Prehypertension, a state between normotension and hypertension, is a strong predictor of hypertension^1^. According to the Seventh Report of the Joint National Committee (JNC-7) guideline, prehypertension is characterized by a systolic blood pressure (SBP) ranging from 120 mm Hg to 139 mm Hg and/or a diastolic blood pressure (DBP) ranging from 80 mm Hg to 90 mm Hg^1^. The prevalence of prehypertension is rapidly increasing worldwide. In the InterASIA study, the prevalence of prehypertension is 21.9% among Chinese adults (25.7% in males and 18.0% in females)^2^, and prehypertension is emerging as an independent risk factor for cardiometabolic disorders, including metabolic syndrome, diabetes, chronic kidney disease, stroke, and cardiovascular diseases^3,4^.

Excessive dietary salt intake plays an important role in the onset and maintenance of hypertension, whereas restricted salt intake lowers blood pressure (BP)^5^. Dietary salt intake by itself, even without causing hypertension or volume overload, might be deleterious, resulting in cardiac remodeling, renal fibrosis, and left ventricular hypertrophy^6–8^. Several mechanisms, including endothelial dysfunction, oxidative stress, inflammation, insulin resistance, and neurogenically mediated increase in peripheral resistance, contribute to the harmful effects of dietary salt^9,10^. Recent studies have shown that increased salt intake may be associated with the pathogenesis of prehypertension^11–13^. However, data on the association between dietary salt intake and prehypertension are lacking.

Uric acid (UA) is the metabolic end product of purine degradation in humans; xanthine oxidase is the enzyme responsible for UA production and free radical damage^14^. Epidemiological studies have identified serum UA is an important risk factor for cardiovascular disease and hypertension^15–17^. For example, Puddu *et al*.^16,17^ found that serum UA could predict not only short-term but also long-term incidence of cardiovascular events as well as cardiovascular death and all-cause mortality. Recent studies have shown that increased sodium intake significantly lowers serum UA^18,19^. However, no research has focused on the relationship between dietary salt intake and UA levels, especially urinary UA excretion, in prehypertensive participants. Furthermore, the relationship between UA and prehypertension and the synergistic effects of UA and dietary salt intake on the risk of prehypertension remain unclear to date.

In the present study, we used our previously established cohort that has been followed up for 30 years to examine the possible associations between urinary sodium excretion, which was used as surrogate for salt intake, and serum and urinary UA levels in prehypertensive participants. We particularly sought to investigate the interactions between urinary sodium excretion and serum UA on the risk of prehypertension in Chinese young adults.

## Methods

### Cohort of study

In March and April 1987, we established the cohort of Hanzhong Adolescent Hypertension Study based on a baseline survey of 4623 adolescents aged 6–15 years in over 20 schools of three towns (Qili, Laojun, and Shayan) in Hanzhong, Shaanxi, China^20,21^. To explore the BP trajectory and its risk factors from children to adults, we made the long-term follow-ups of this cohort in 1989, 1992, 1995, 2005, 2013, and 2017 (Supplementary Figure S1).

**Figure 1:**
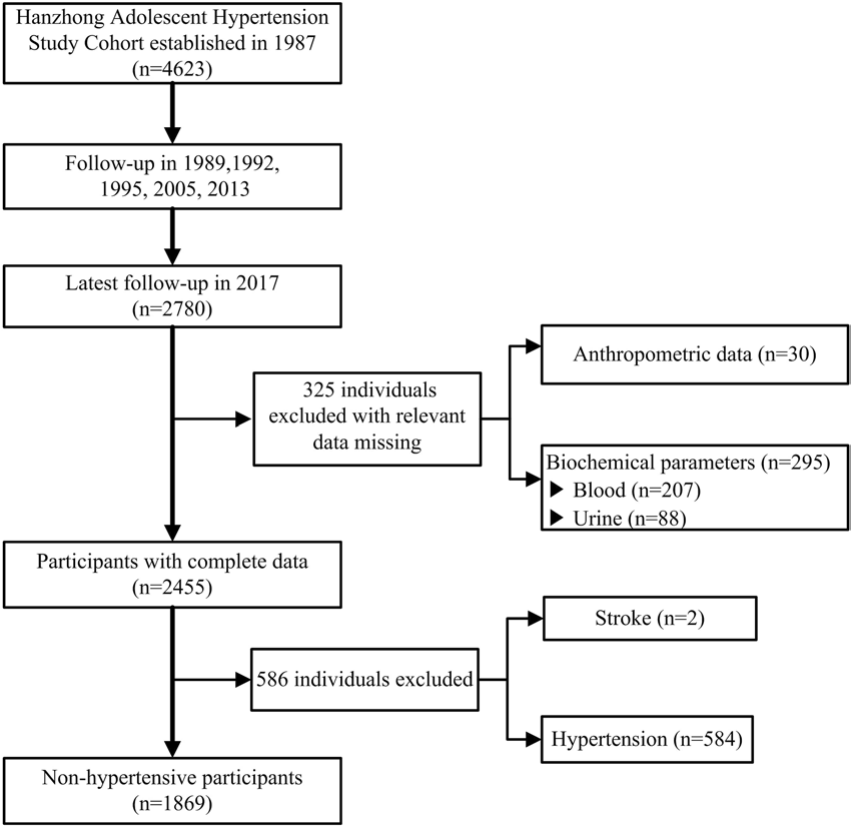
Flow diagram showing the selection of the study population.

In this study, we followed up this cohort from April to July 2017, and a total of 2780 were followed up this time. The total rate of this follow-up was 60.3%, which was very rare for such a long-term follow-up. The participant selection process is described in Fig. 1. Of the 2780 participants, 911 were excluded from the current analysis for the following reasons: hypertension defined as a SBP ≥140 mm Hg or DBP ≥90 mm Hg or current use of antihypertensive medications (n = 584), missing important data (BP, n = 29; height and weight, n = 1; blood biochemistry, n = 207; urinary biochemistry, n = 87; urinary creatinine, n = 1), and self-identified history of stroke (n = 2). The remaining 1869 individuals were included in the analysis. Data including social demographic survey (age, gender, education, occupation, medical conditions, and prescription and nonprescription medication use), physical activity, physical examination with anthropometric measurements, and laboratory testing were collected by trained physicians or medical students.

The present study complied with the Declaration of Helsinki, and the research protocol was approved by the Ethics Committee of the First Affiliated Hospital of Xi’an Jiaotong University (code: 2015–128). Upon admission, all subjects provided their informed consent. Trial Registration Number: NCT02734472 (http://www.clinicaltrials.gov). Date of registration: 12/04/2016.

### BP measurement

BP was measured by six trained staff members using a standard mercury sphygmomanometer as previously described^22,23^. The average of three readings was recorded. Study participants were classified into one of the two non-hypertensive BP categories in accordance with the criteria of the Joint National Committee on Prevention, Detection, Evaluation, and Treatment of High Blood Pressure and the International Society of Hypertension: prehypertension (SBP of 120–140 mm Hg or DBP of 80–90 mm Hg) and normotension (SBP <120 mm Hg and DBP <80 mm Hg).

### Blood biochemical analyses

Blood samples were obtained by peripheral venous puncture, immediately centrifuged at 3000 × *g* for 10 min, and then stored at −80 °C until analysis. Total cholesterol, triglyceride, high-density lipoprotein (HDL), low-density lipoprotein (LDL), serum creatinine, and blood glucose were measured using an automatic biochemical analyzer (model 7600; Hitachi, Ltd., Tokyo, Japan). Serum UA level was measured with a Hitachi clinical chemistry analyzer with the uricase HMMPS method. Five samples were used to evaluate the intra-assay and inter-assay coefficients of variation (CV), which ranged from 2.3% to 4.5% and from 3.2% to 6.4%, respectively. Estimated glomerular filtration ratio (eGFR) was calculated using the Modification of Diet in Renal Disease formula^24^. Hyperuricemia was defined as serum UA level of ≥420 μmol/L for men and ≥360 μmol/L for women.

### Urinary biochemical testing

A morning fasting midstream urine sample was collected from each participant and frozen at −20 °C to −40 °C. All urine samples were shipped in ambient packaging with the use of ice boxes to the clinical laboratory at the First Affiliated Hospital of Xi’an Jiaotong University in Xi’an, China. Urinary sodium, creatinine, UA, and albumin were measured by an automatic biochemical analyzer (Hitachi, Ltd., Japan) at a certified clinical lab. The intra- and inter-assay CVs were 0.42% and 1.69% for urinary sodium, 1.25% and 2.17% for urinary creatinine, 0.64% and 2.13% for urinary albumin, and 3.1% and 5.2% for urinary UA, respectively. The Kawasaki formula was used to estimate 24-hour urinary sodium excretion, and the estimate was used as surrogate for salt intake^25,26^. A brief description of the validation of the Kawasaki formula is provided in the article by Mente *et al*.^27^.

### Statistical analysis

Data are expressed as means ± standard deviation (SD) for normally distributed values, as median (25th and 75th percentile) for non-normally distributed values, and as percentages. The Kolmogorov–Smirnov test was used to determine whether the continuous variables were normally distributed. Statistical significance of difference among the groups was calculated using χ2-test for categorical variables, Student’s t-test for continuous variables in normally distributed data, and Mann–Whitney test for non-normally distributed data. One-way ANOVA was employed to compare the mean values across the sodium excretion quartile groups. Multiple linear regression and Pearson correlation analyses were performed to determine the strength of association between serum and urinary UA levels and other continuous parameters. The adjusted odds ratio (OR) and 95% confidence interval (CI) were calculated to estimate the risk for prehypertension of single/combined status of sodium excretion and serum UA, and quartiles of sodium excretion and serum UA by logistic regression analyses. All statistical analyses were performed using SPSS for Windows version 16.0 (SPSS, Inc., Chicago, IL). A two-sided *P* < 0.05 was considered statistically significant.

### Data availability

All raw experimental data used in this study is available from the corresponding authors on request.

## Results

### Characteristics of study participants

A total of 1869 individuals who met the inclusion criteria were finally followed up and included in the current analyses. Of these 1869 individuals, 842 (45.1%) were identified as prehypertensive while 1027 (54.9%) as normotensive.

Firstly, a total of 842 prehypertensive subjects were analyzed to examine the association between sodium excretion and serum and urinary UA levels. Table 1 presents the characteristics of the prehypertensive participants according to the quartiles of urinary sodium excretion. Individuals with higher sodium excretion were tended to be male, younger and fatter; unwilling to engage in physical activity; and usually possessed lower HDL-C and serum creatinine but higher eGFR and urinary albumin/creatinine. It was noteworthy that starting with the lowest quartile of estimated sodium excretion, the excretion of urinary UA/creatinine was 0.12, 0.17, 0.24, and 0.33 for increasing quartiles, respectively (*P* for trend <0.001). No significant difference was observed in serum UA across each quartile of urinary sodium excretion in prehypertensive participants (*P* for trend = 0.951).

**Table 1:** Baseline characteristics according to salt intake in prehypertensive subjects (n = 842).

Characteristics	Quartiles of Sodium excretion (g/day)				
	I (<4.19)	II (4.19−5.14)	III (5.14−6.07)	IV (>6.07)	*P* for trend
No. of subjects	210	210	210	212	−
Age (years)	44 (40.0–45.0)	43.5 (41.0–45.0)	44.0 (40.8–45.0)	42.0 (39.0–45.0)	0.025
Gender (M/F)	110/100	128/82	144/66	152/60	<0.001
Body mass index (kg/m^2^)	23.7 ± 3.0	24.5 ± 2.9	24.8 ± 2.9	24.8 ± 3.0	<0.001
Alcohol consumption (n, %)	62 (29.5)	71 (33.8)	68 (32.4)	79 (37.3)	0.133
Current smoking (n, %)	84 (40.0)	80 (38.1)	91 (43.3)	100 (47.2)	0.079
Diabetes mellitus (n, %)	8 (3.8)	3 (1.4)	7 (3.3)	6 (2.8)	0.841
Heart rate (beats/min)	75.0 (69.0–82.2)	73.0 (66.0–79.0)	73.0 (65.0–78.0)	72.0 (65.0–77.0)	0.011
**Level of physical activity (n**, **%)**					
Almost no	65 (31.0)	92 (43.8)	110 (52.4)	102 (48.1)	0.006
Light	124 (59.0)	102 (48.6)	85 (40.5)	102 (48.1)	0.059
Moderate	13 (6.2)	12 (5.7)	7 (3.3)	5 (2.4)	0.184
Heavy	8 (3.8)	4 (1.9)	8 (3.8)	3 (1.4)	0.307
Systolic blood pressure (mmHg)	125.0 (121.7–130.0)	126.3 (122.3–130.8)	126.5 (123.3–131.4)	126.3 (122.7–131.0)	0.399
Diastolic blood pressure (mmHg)	80.0 (75.3–83.3)	79.3 (74.3–83.3)	80.2 (75.7–84.0)	80.3 (76.7–84.0)	0.6
Serum uric acid (μmol/L)	288.2 ± 81.8	297.5 ± 87.6	295.2 ± 69.7	289.5 ± 76.7	0.951
Fasting glucose (mmol/L)	4.58 (4.29–4.93)	4.58 (4.28–4.96)	4.60 (4.31–4.93)	4.66 (4.35–5.00)	0.479
Total cholesterol (mmol/L)	4.64 ± 0.81	4.61 ± 0.81	4.66 ± 0.84	4.49 ± 0.75	0.106
Triglycerides (mmol/L)	1.31 (0.94–1.85)	1.47 (1.03–2.17)	1.46 (1.07–2.11)	1.47 (1.02–2.18)	0.216
LDL- cholesterol (mmol/L)	2.59 ± 0.66	2.58 ± 0.61	2.65 ± 0.68	2.45 ± 0.62	0.072
HDL- cholesterol (mmol/L)	1.19 (0.99–1.37)	1.12 (0.97–1.31)	1.22 (0.97–1.28)	1.11 (0.93–1.30)	0.025
Serum creatinine (μmol/L)	79.2 ± 13.8	77.1 ± 14.6	78.1 ± 13.0	75.2 ± 13.9	0.027
eGFR (mL/min/1.73 m^2^)	65.8 (60.3–73.8)	68.1 (61.3–76.4)	69.8 (63.1–78.1)	73.9 (66.0–81.5)	<0.001
Urine albumin/creatinine (mg/g)	8.02 (5.30–13.19)	8.05 (5.46–12.56)	9.33 (5.88–15.62)	10.75 (6.32–19.51)	<0.001
Urinary uric acid/creatinine	0.12 (0.08–0.19)	0.17 (0.12–0.24)	0.24 (0.15–0.36)	0.33 (0.20–0.47)	<0.001
Urinary sodium excretion (g/day)	3.62 (3.22–3.92)	4.66 (4.43–4.91)	5.54 (5.32–5.78)	6.84 (6.38–7.43)	<0.001

LDL, low-density lipoprotein; HDL, high-density lipoprotein; eGFR, estimated glomerular filtration rate; Non-normally distributed variables are expressed as the median (interquartile range). All other values are expressed as mean ± SD or n, %.

### Association between salt intake and serum and urinary UA levels in prehypertensive participants

**Figure 2:**
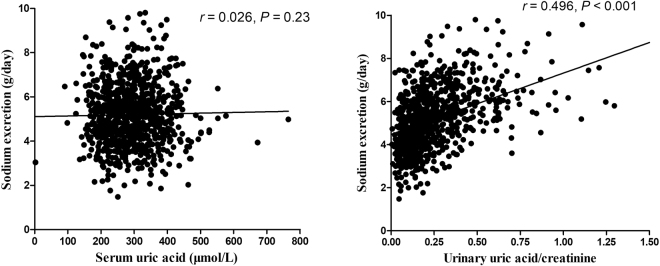
The correlations between estimated sodium excretion and serum UA (**a**) and urinary UA/creatinine excretions (**b**) in prehypertensive participants.

Estimated sodium excretion, the substitute for salt intake, positively correlated with urinary UA/creatinine excretions (*r* = 0.496, *P* < 0.001) but not with serum UA (*r* = 0.026, *P* = 0.23) in the prehypertensive group (Fig. 2). In multiple linear regression analysis, we assessed the potential confounding factors [age, gender, body mass index (BMI), fasting glucose, total cholesterol, triglycerides, LDL, HDL, serum creatinine, alcohol consumption, smoking status, diabetes, and physical activity] for serum and urinary UA in the prehypertensive participants. In this model, urinary UA/creatinine positively correlated with estimated sodium excretion (*β* = 0.488, *P* < 0.001) and gender (*β* = 0.27, *P* < 0.001).

In addition, serum UA levels positively correlated with serum creatinine, BMI, total cholesterol, and alcohol consumption but negatively correlated with gender, HDL, and fasting glucose. However, no correlation was found between serum UA and urinary sodium excretion in the prehypertensive group (Supplementary Table S1). After adjustment for various confounders, BMI and serum creatinine but not sodium excretion (*OR* = 0.96, *P* = 0.393; Supplementary Table S2) were associated with a higher risk of hyperuricemia in the prehypertensive participants.

### Individual effects of salt intake and serum UA on prehypertension

Next, to examine the relationship between salt intake and serum UA, and the risk of prehypertension, we include the normotensive (n = 1027) and prehypertensive (n = 842) subjects in the current analysis. Table 2 shows the characteristics of all subjects according to BP status. Prevalence of men, alcohol consumption, smoking, light physical activity, and age, BMI, SBP, DBP, total cholesterol, fasting glucose, triglycerides, LDL, serum UA, serum creatinine, eGFR, urinary albumin/creatinine and sodium excretions were higher in participants with prehypertension than those with normotension, but HDL-C was higher in those with normotension. There were no inter-group differences regarding prevalence of diabetes mellitus and urinary UA/creatinine excretions. Furthermore, sodium excretion [1.06 (1.03–1.09)] and serum UA [1.003 (1.001–1.004)] were significantly associated with the risk of prehypertension after adjusting multiple confounders. Urinary UA/creatinine (*P* = 0.107) did not remain in the final model (Table 3).

**Table 2:** Characteristics of participants categorized by blood pressure status (n = 1869).

Characteristics	All	Normotension	Prehypertension	*P*-value
No. of subjects	1869	1027	842	—
Age (years)	43.0 (40.0–45.0)	42.0 (39.0–45.0)	43.0 (40.0–45.0)	<0.001
Gender (M/F)	951/918	417/610	534/308	<0.001
Body mass index (kg/m^2^)	23.3 (21.5–25.4)	22.7 (21.0–24.5)	24.3 (22.4–26.4)	<0.001
Alcohol consumption (n, %)	475 (25.4)	195 (19.0)	280 (33.3)	<0.001
Current smoking (n, %)	654 (35.0)	299 (29.1)	355 (42.2)	<0.001
Diabetes mellitus (n, %)	43 (2.3)	19 (1.9)	24 (2.9)	0.101
**Level of physical activity (n**, **%)**				
Almost no	755 (40.5)	387 (37.7)	368 (43.8)	0.489
Light	981 (52.6)	568 (55.4)	413 (49.2)	<0.001
Moderate	78 (4.2)	41 (4.0)	37 (4.4)	0.651
Heavy	51 (2.7)	30 (2.9)	21 (2.5)	0.208
Heart rate (beats/min)	72.0 (66.0–79.0)	72.0 (66.0–79.0)	73.0 (66.0–79.0)	0.115
Systolic blood pressure (mmHg)	117.6 ± 10.5	110.0 ± 6.8	126.9 ± 5.4	<0.001
Diastolic blood pressure (mmHg)	73.2 ± 8.1	68.2 ± 6.0	79.4 ± 5.7	<0.001
Serum uric acid (μmol/L)	271.1 (219.2–322.2)	255.6 (211.6–305.0)	290.0 (233.6–340.6)	<0.001
Fasting glucose (mmol/L)	4.54 (4.25–4.86)	4.47 (4.21–4.79)	4.60 (4.31–4.95)	<0.001
Total cholesterol (mmol/L)	4.50 (4.02–4.99)	4.46 (3.97–4.91)	4.55 (4.06–5.08)	<0.001
Triglycerides (mmol/L)	1.26 (0.92–1.84)	1.17 (0.86–1.62)	1.41 (1.01–2.06)	<0.001
LDL- cholesterol (mmol/L)	2.49 (2.11–2.88)	2.45 (2.05–2.80)	2.54 (2.15–2.98)	<0.001
HDL- cholesterol (mmol/L)	1.16 (1.00–1.35)	1.19 (1.03–1.38)	1.13 (0.97–1.31)	<0.001
Serum creatinine (μmol/L)	75.6 ± 13.8	74.1 ± 13.4	77.5 ± 13.9	<0.001
eGFR (mL/min/1.73 m^2^)	68.3 (61.9–76.5)	67.9 (61.4–75.6)	69.0 (62.4–77.6)	0.034
Urine albumin/creatinine (mg/g)	8.02 (5.30–13.19)	7.57 (4.96–12.16)	8.59 (5.76–14.97)	<0.001
Urinary uric acid/creatinine	0.20 (0.12–0.33)	0.20 (0.12–0.33)	0.19 (0.12–0.33)	0.618
Urinary sodium excretion (g/day)	5.01 ± 1.37	4.86 ± 1.34	5.20 ± 1.38	<0.001

LDL, low-density lipoprotein; HDL, high-density lipoprotein; eGFR, estimated glomerular filtration rate; Non-normally distributed variables are expressed as the median (interquartile range). All other values are expressed as mean ± SD or n, %.

**Table 3:** Association between various characteristics and prehypertension by stepwise multiple logistic regression analysis (n = 1869).

Characteristics	Odds Ratios (confidence interval)	*P* value
Gender (Male)	1.47 (1.14–1.89)	0.003
Age (years)	1.06 (1.03–1.09)	<0.001
Alcohol consumption (%)	1.41 (1.10–1.80)	0.007
Fasting glucose (mmol/L)	1.11 (1.02–1.22)	0.02
Triglycerides (mmol/L)	1.23 (1.11–1.37)	<0.001
LDL-Cholesterol (mmol/L)	1.20 (1.03–1.40)	0.022
Sodium excretion (g/day)	1.06 (1.03–1.09)	<0.001
Serum uric acid (μmol/L)	1.003 (1.001–1.004)	0.002

Logistic regression analyses were used to test the risk of hyperuricemia, after adjustment for age, gender, BMI, fasting glucose, total cholesterol, triglycerides, LDL, HDL, serum creatinine, alcohol consumption, smoking status, diabetes and physical activity. Urinary UA/creatinine (*P* = 0.107) did not remain in the final model.

We further assessed the individual effects of salt intake and serum UA on prehypertension risk (Table 4). In an age- and sex-adjusted model, the ORs (95% CI) of prehypertension across increasing quintiles of sodium excretion were 1.00, 1.16 (0.88–1.52), 1.42 (1.08–1.86) and 1.70 (1.29–2.24) (*P* for trend <0.001). In the multivariate model, further adjusting for BMI, fasting glucose, total cholesterol, triglycerides, LDL, HDL, serum creatinine, alcohol consumption, smoking status, diabetes and physical activity, the ORs (95% CI) were 1.00, 1.15 (0.87–1.52), 1.46 (1.11–1.91) and 1.68 (1.27–2.22) (*P* for trend = 0.001). The effect of serum UA on the risk of prehypertension was also estimated by the quartiles of serum UA. The quartiles of serum UA were defined as follows: quartile 1: < 219.2 μmol/L; quartile 2: 219.2–271.1 μmol/L; quartile 3: 271.1–322.2 μmol/L; and quartile 4: > 322.2 μmol/L. After adjustment for multiple confounders, compared with the lowest serum UA quartile, the ORs of prehypertension were 1.22 (95% CI, 0.92–1.63), 1.46 (95% CI, 1.07–1.99) and 1.71 (95% CI, 1.21–2.42); *P* = 0.019 for trend.

**Table 4:** Association between each quartile of urinary sodium excretion and serum UA and incidence of prehypertension (n = 1869).

	Nomotensive controls	Prehypertensive cases	Odds Ratios (95% confidence interval)	
			Age, sex-adjusted	Multivariate*
**Urinary sodium excretion**				
Quartile 1 (<4.02 g/day)	293 (62.6%)	175 (37.4%)	1.00 (reference)	1.00 (reference)
Quartile 2 (4.02−4.93 g/day)	269 (58.0%)	195 (42.0%)	1.16 (0.88–1.52)	1.15 (0.87–1.52)
Quartile 3 (4.93−5.88 g/day)	242 (51.8%)	225 (48.2%)	1.42 (1.08–1.86)	1.46 (1.11–1.91)
Quartile 4 (>5.88 g/day)	223 (47.4%)	247 (52.6%)	1.70 (1.29–2.24)	1.68 (1.27–2.22)
P for trend	0.012	0.002	<0.001	0.001
**Serum uric acid**				
Quartile 1 (<219.2 μmol/L)	321 (68.7%)	146 (31.3%)	1.00 (reference)	1.00 (reference)
Quartile 2 (219.2−271.1 μmol/L)	279 (59.9%)	187 (40.1%)	1.27 (0.96–1.68)	1.22 (0.92–1.63)
Quartile 3 (271.1−322.2 μmol/L)	235 (50.1%)	234 (49.9%)	1.60 (1.18–2.16)	1.46 (1.07–1.99)
Quartile 4 (>322.2 μmol/L)	192 (41.1%)	275 (58.9%)	2.04 (1.47–2.83)	1.71 (1.21–2.42)
*P* for trend	<0.001	<0.001	<0.001	0.019

Logistic regression analyses were used to test the risk of prehypertension, after adjustment for age, gender, BMI, fasting glucose, total cholesterol, triglycerides, LDL, HDL, serum creatinine, alcohol consumption, smoking status, diabetes and physical activity.

### Synergistic effect of salt intake and serum UA on prehypertension

**Figure 3:**
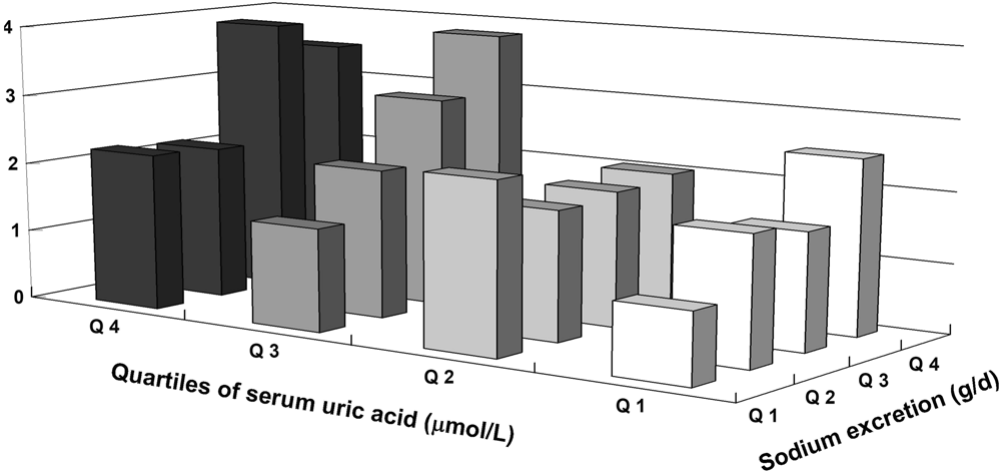
Synergistic effect of salt intake and serum UA on the risk of prehypertension. The ORs were compared with a common reference group (the lowest quartile of sodium excretion and the lowest quartile of serum UA).

Finally, the synergistic effects of salt intake and serum UA on the risk of prehypertension were estimated after controlling for confounders (Fig. 3 and Supplementary Table S3). A formal test for interaction between sodium excretion and serum UA revealed a significant interaction (*P* = 0.046). The ORs of prehypertension were about 3.48 times as much as in the highest sodium excretion and serum UA quartiles (sodium excretion >5.88 g/day and serum UA >322.2 µmol/L) than in the lowest sodium excretion and serum UA quartiles (sodium excretion <4.02 g/day and serum UA <219.2 µmol/L).

## Discussion

In this cohort of Chinese individuals with prehypertension, urinary sodium excretion showed no association with serum UA but significantly correlated with urinary UA. To the best of our knowledge, this study is the first to examine the relationship of salt intake to serum and urine UA levels in prehypertensive subjects.

Although the relationship between salt intake and hypertension is well established, its relationship with UA remains controversial. One proposed hypothesis states that higher UA levels represent an evolutionary advantage in *Homo sapiens*, allowing them to maintain BP when access to sodium is scarce^28^. This theory was demonstrated in uricase-deficient rat models showing an increase in BP from hyperuricemia in the context of a low-salt diet^29^. Additional rat models have supported this hypothesis, showing that UA upregulates and activates epithelial sodium channels in nephrons^30^. Hou *et al*.^31^ have recently reported in a cross-sectional study of 1668 Chinese participants that high salt intake enhances associations of blood UA with hypertension and related cardiovascular risk. However, limited studies focused on salt intake and UA. One large longitudinal cohort study conducted in 4062 participants from Netherlands found that higher sodium intake predicted lager longitudinal increase of serum UA. It was reported that each 1 gram higher sodium intake was associated with a 1.4 μmol/L increase in serum UA^32^. Another trial performed in 27 men showed that increasing sodium intake from 20 mEq/day to 200 mEq/day decreased UA levels by 1 mg/dL^33^. In addition, a randomized crossover trial of 103 adults with prehypertension or stage I hypertension showed that 30 days of low versus high sodium intake (60 versus 180 mmol/day) significantly decreased serum UA^18,19^. In this study, we showed that the serum UA levels were similar between each quartile of estimated sodium excretion in prehypertensive subjects. Furthermore, sodium excretion was not correlated with serum UA and hyperuricemia in the unadjusted and adjusted analyses. The discrepant results of these studies may be attributed to their different study populations, designs, sample sizes, and racial differences.

UA is a product of the metabolic breakdown of purine nucleotides. Approximately 70% of UA is excreted into the urine but is easily filtered into the renal tubule, and about 90% of filtered UA is reabsorbed by the S1 segment of the proximal convoluted tubule^32^. Approximately 10% of filtered UA is excreted^34^. To the best of our knowledge, this present study is the first to demonstrate that urinary excretions of UA were significantly associated with sodium excretion in prehypertensive subjects. The mechanism by which sodium intake increases urinary excretion of UA remains unclear. It is possible that the relationship between sodium intake and urinary urate excretion results from effects of sodium intake on glomerular filtration rate and excretion or absorption of urate. Previous physiology studies have shown that reabsorption of sodium and urate accompanies one another at different sites in the nephron^35,36^. Thus, it is possible that decreased renal reabsorption of sodium from excess sodium intake contributes to a decrease in urate reabsorption. This hypothesis has been evidenced by our interventional study showing that urinary UA excretions were markedly increased during high-salt intake, which was further reinforced by the observation that urinary UA positively correlated with urinary sodium excretion^37^. Furthermore, a Spanish study also found a directly correlation between the clearance of UA and fractional excretion of sodium, indicating the potential interaction of sodium and UA excretion^38^. Finally, this relationship may reflect action of the renin-angiotensin system, as uric acid is inversely related to vascular resistance^39^ and renal blood flow^40^. Similarly, angiotensin II has been shown to decrease urate excretion after an acute infusion^41,42^. Determining the molecular mechanism and signaling molecules responsible for the effects of salt intake on urinary UA can be of great interest.

A limited number of studies have examined the relationship serum UA and prehypertension, and findings are conflicting. One US study found a positive association between serum UA and prehypertension with an OR of 1.96 for the top category of serum UA levels compared with the lowest^43^. Jiang *et al*.^44^ described that the OR for prehypertension is 1.36 in subjects with UA ≥365 μmol/L compared with those with UA <215.9 μmol/L after adjusting for many confounders. The other two cross-sectional studies demonstrated that serum UA was independently related to the prevalence of prehypertension in Chinese adults^45,46^. In contrast, Vucak *et al*.^47^ determined that no association existed between elevated serum UA level and prehypertension; this might be because of higher background rate of prehypertension with increasing age that would contribute to a reduction in the odd ratios for serum UA. Recently, a prospective cohort study demonstrates that serum UA is an independent predictor for developing prehypertension^48^. In addition, Soletsky *et al*.^49^ reported that UA reduction rectifies prehypertension in obese adolescents. In the present study, we consistently showed that higher serum UA category was significantly associated with an increased OR for the presence of prehypertension, compared with the reference group. And the observed positive association between serum UA and prehypertension consistently occurred when serum UA was considered as a continuous variable.

Previously, ample evidence suggests that excess salt intake is positively associated with elevated blood pressure and it can be lowered with reductions in dietary salt^5^. However, clinical trials scarcely examined the relationship between salt intake and prehypertension. Moinuddin *et al*.^11^ showed that daily salt intake of prehypertensives (21.2 ± 1.2 g/day) was significantly greater than normotensive subjects (9.0 ± 0.5 g/day). This result is similar to our study, which found that compared with participants without prehypertension, those with prehypertension tended to have higher urinary sodium excretions (5.20 ± 1.38 *vs*. 4.86 ± 1.34; *P* < 0.001). We further observed that the risk of prehypertension was significantly increased with the increasing quartiles of sodium excretion. In addition, Forman *et al*.^32^ in a large, prospective, population-based cohort, found that a higher sodium intake is associated with an increased risk of developing hypertension, particularly in those individuals who have higher levels of serum UA. Our results also showed that salt intake significantly interacted with serum UA. Taken both serum UA and sodium excretion into consideration to assess prehypertension, we found that the risk of prehypertension in the highest quartiles of serum UA and sodium excretion was 3.48 (95% CI, 3.32–5.86) times greater than in the lowest quartile. In other words, participants with higher serum UA levels and salt intake simultaneously were more likely to have a higher risk for prehypertension.

This study has limitations that deserve mention. Firstly, since the study population was included from our previously established cohort, all participants in the present work were middle aged and youth between the ages of 35 and 48 years during the follow-up at 2017. In addition, 24-h urinary sodium excretion was estimated using spot urine samples. Estimated sodium excretions may change according to the urine sampling time because urinary sodium excretion has a circadian rhythm and may also be influenced by the time at which food is consumed. Thus, a single measurement may be insufficient to assess the sodium excretion of individuals. However, spot urine samples are practical at general medical facilities, and the reliability of the findings obtained may be improved using a calculation formula incorporating the estimated 24-hour urinary creatinine excretion based on age, height, and body weight. The validation of the Kawasaki formula was conducted by Mente *et al*.^27^. Finally, the study was a single-center, cross-sectional study. A multi-center, prospective trial will be conducted to further understand and confirm the conclusions.

In conclusion, the present study showed that urinary sodium excretion was significantly associated with urinary UA excretions in prehypertensive individuals. However, we failed to find a significant relationship between sodium excretion and serum UA in this Chinese population. In addition, elevated serum UA and sodium excretions appeared to be associated with the development of prehypertension. Individuals with higher serum UA levels and sodium excretions simultaneously had a higher risk for prehypertension. Further clinical trials that include participants with hypertension to investigate evaluate the joint effects of salt intake and serum UA on hypertension and other cardiovascular diseases can be of significant interest. Our findings supported the need for the development of a salt reduction programme.

## Electronic supplementary material

Supplementary Information
