# Supplementary material for: Association between urinary sodium excretion and uric acid, and its interaction on the risk of prehypertension among Chinese young adults
Source: Sci Rep. 2018 May 17;8:7749. doi: 10.1038/s41598-018-26148-3 (PMC5958063; doi:10.1038/s41598-018-26148-3)
Supplement: Supplementary file 1 — Supplementary Information [file 41598_2018_26148_MOESM1_ESM.pdf]

Association between urinary sodium excretion and uric acid, and its interaction on the risk of prehypertension among Chinese young adults

Yang Wang [1,2†], Jia-Wen Hu [1,2†], Peng-Fei Qu [3†], Ke-Ke Wang [1,2], Yu Yan [1,2], Chao Chu [1,2], Wen-Ling Zheng [1,2], Xian-Jing Xu [4], Yong-Bo Lv [1,2], Qiong Ma [1], Ke Gao [1], Yue Yuan [1], Li Hao [5], Zu-Yi Yuan [1,2], Jian-Jun Mu [1,2*]

1. Department of Cardiology, First Affiliated Hospital of Medical School, Xi’an Jiaotong University, Xi’an, China;
2. Key Laboratory of Molecular Cardiology of Shaanxi Province, Xi’an, China;
3. Assisted Reproduction Center, Northwest Women and Children’s Hospital, Xi'an, China;
4. General Ward, Henan Provincial People’s Hospital, Zhengzhou, Henan, China.
5. Department of Critical Care Medicine, First Affiliated Hospital of Medical School, Xi’an Jiaotong University, Xi’an, China;

† These authors contributed equally to this work.

* Correspondence author: Jian-Jun Mu, MD

Address: Department of Cardiology, First Affiliated Hospital of Medical School, Xi’an Jiaotong University, 277 Yanta West Street, Xi’an, 710061, China.

E-mail : mujjun@163.com

Phone: 86-29-85323804

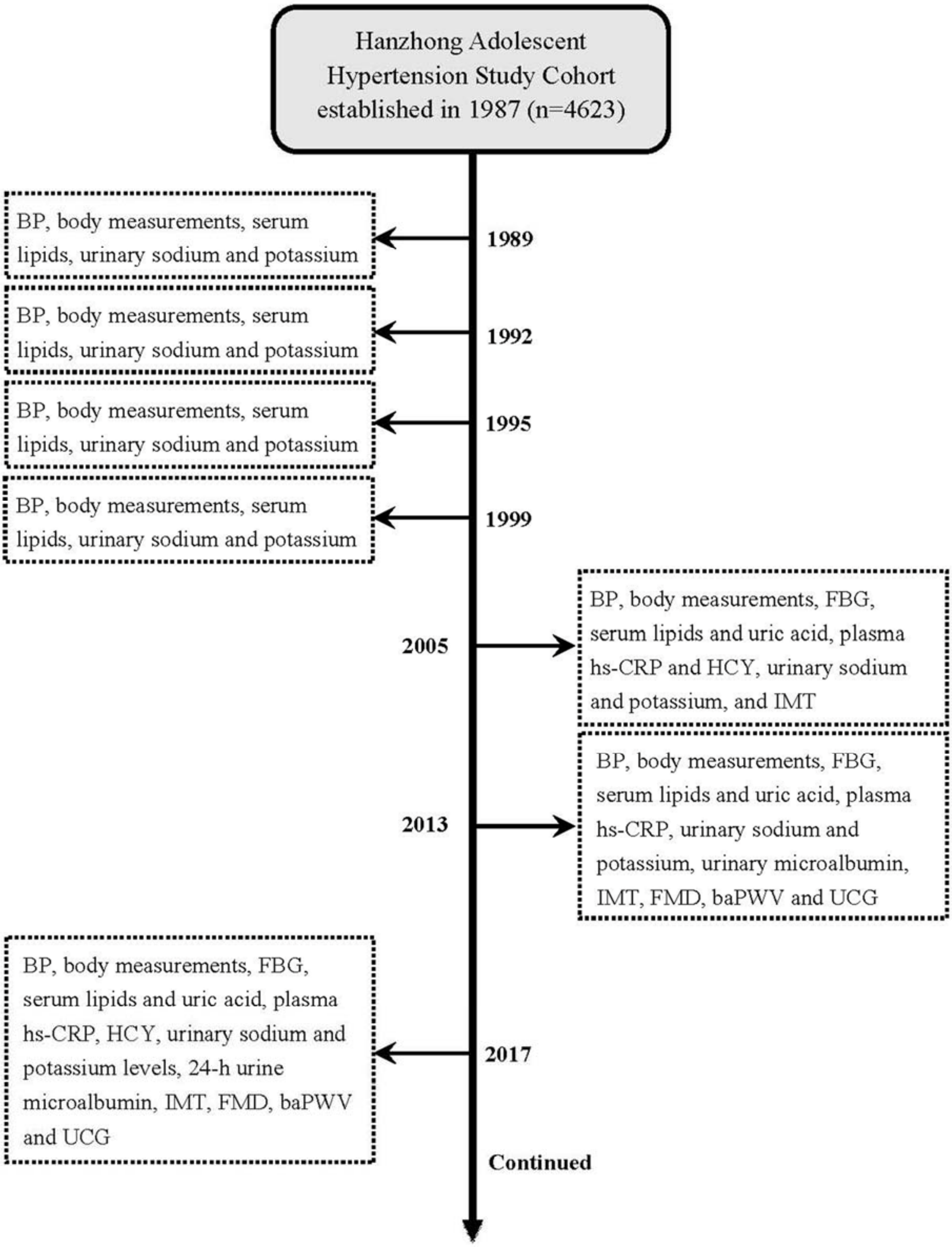

Figure S1. The protocol of longitudinal follow-up of the cohort. BP, blood pressure; FBG, fasting blood glucose; hs-CRP, high-sensitivity C-reactive protein; HCY, homocysteine; IMT, intima-media thickness; FMD, flow-mediated dilation; baPWV, brachial-ankle pulse wave velocity; UCG, ultrasonic cardiogram.

Table S1. Relationship between serum UA levels and various characteristics by multiple linear regression analysis in prehypertensive subjects (n=842).

Characteristics	β	*P* value
Serum creatinine (μmol/L)	0.32	<0.001
Gender	-0.282	<0.001
HDL- cholesterol (mmol/L)	-0.133	<0.001
Body mass index (kg/m^2)	0.122	<0.001
Fasting glucose (mmol/L)	-0.08	0.004
Total cholesterol (mmol/L)	0.077	0.008
Alcohol consumption (%)	0.066	0.026
Sodium excretion (g/day)	-0.031	0.269

Logistic regression analyses were used to test the risk of prehypertension, after adjustment for age, BMI, FPG, CHO, TG and serum UA as well as smoking status and alcohol consumption.

Table S2. Association between hyperuricemia and various characteristics by stepwise multiple logistic regression analysis in prehypertensive subjects (n = 842).

Characteristics	Odds Ratios (confidence interval)	*P* value
Body mass index (kg/m^2)	1.16 (1.05-1.27)	0.003
Serum creatinine (μmol/L)	1.06 (1.04-1.08)	<0.001
Sodium excretion (g/day)	0.96 (0.87-1.05)	0.393

Logistic regression analyses were used to test the risk of hyperuricemia, after adjustment for age, gender, BMI, fasting glucose, total cholesterol, triglycerides, LDL, HDL, serum creatinine, alcohol consumption, smoking status, diabetes and physical activity. Sodium excretion (*OR*=0.96, *P* = 0.393) did not remain in the final model.

Table S3. Odds ratios for the joint effect of salt intake and serum UA on incidence of prehypertension

Serum UA (μmol/L)	Sodium excretion (g/day) Adjusted OR (95% CI) in all participants (n=1869)			
	Q1 (<4.02)	Q2 (4.02–4.93)	Q3 (4.93–5.88)	Q4 (>5.88)
Q1 (<219.2)	1.00 (reference)	1.81 (1.01-3.23)	1.66 (0.89-3.08)	2.49 (1.38-4.48)
Q2 (219.2–271.1)	2.41 (1.36-4.26)	1.83 (1.00-3.36)	1.92 (1.07-3.45)	2.02 (1.11-3.69)
Q3 (271.1–322.2)	1.46 (0.80-2.65)	2.12 (1.15-3.89)	2.99 (1.64-5.43)	3.81 (2.07-7.02)
Q4 (>322.2)	2.24 (1.18-4.25)	2.18 (1.19-4.01)	3.90 (2.10-7.24)	3.48 (1.87-6.48)

OR, odds ratio; CI, confidence interval; UA, uric acid. Adjusted for age, gender, BMI, fasting glucose, total cholesterol, triglycerides, LDL, HDL, serum creatinine, alcohol consumption, smoking status, diabetes and physical activity.
